# Implementation of decarbonisation actions in general practice: a systematic review and narrative synthesis protocol

**DOI:** 10.1136/bmjopen-2024-087795

**Published:** 2024-09-16

**Authors:** Florence Karaba, Ana Raquel Nunes, Olivia Geddes, Helen Atherton, Frederik Dahlmann, Abi Eccles, Michael Gregg, Rachel Spencer, Helen Twohig, Jeremy Dale

**Affiliations:** 1 Medical School, University of Warwick, Coventry, UK; 2 Warwick Medical School, University of Warwick, Coventry, UK; 3 University of Southampton, Southampton, UK; 4 Warwick Business School, University of Warwick, Coventry, UK; 5 Warwick Primary Care, University of Warwick, Coventry, UK; 6 PPI co-applicant, UK; 7 Keele University, Keele, UK

**Keywords:** Primary Care, QUALITATIVE RESEARCH, Climate Change

## Abstract

**Abstract:**

**Introduction:**

There is growing recognition of the importance of primary care in addressing climate change. The World Organisation of Family Doctors has urged general practitioners worldwide to commit to tackling climate change and to serve as agents of systemic and individual change. Though an increasing number of resources have become available to support the decarbonisation of primary care, there remains a lack of evidence about how primary care teams are using them, their reach across practices, their level of adoption and maintenance, their cost impact and their effect on carbon emissions. This systematic review aims to understand how primary care, with a focus on general practice or equivalent settings within the context of primary care, is implementing decarbonisation actions to reduce carbon emissions arising from its operations, assess efficacy of the actions and generate recommendations on how to assist and accelerate their implementation and effectiveness.

**Methods and analysis:**

The literature search will be conducted on Medline, Embase, Web of Science, CINAHL and ProQuest, from 2007 to 29 March 2024. Article screening will be based on specified inclusion and exclusion criteria. Narrative synthesis will be used to analyse and integrate findings to offer new insights into key mechanisms that support decarbonisation in general practice and help refine an initial programme theory. The reporting of the systematic review will follow the Preferred Reporting Items for Systematic Review and Meta-Analysis framework.

**Ethics and dissemination:**

This review did not involve the collection or analysis of any data that was not included in previously published research in the public domain. The results will be disseminated through peer-reviewed publication and conference presentations.

**PROSPERO registration number:**

CRD42023470889.

**STRENGTHS AND LIMITATIONS OF THIS STUDY**

- Thorough literature search of five large electronic databases and reporting as per Preferred Reporting Items for Systematic Reviews and Meta-Analyses guidelines.
- The study screening, selection, data extraction and assessment of the risk of bias will be completed by two independent reviewers.
- Search strategy codeveloped with an experienced librarian and customised to databases.
- An English language restriction will be applied in the selection of the studies. This may limit the inclusion of relevant literature and the applicability of the review’s findings.
- The certainty of the evidence of the systematic review may be undermined by the limited number of studies available on the subject.

## Background

 The climate emergency is contributing to a health crisis.^1^ It brings substantial health effects related to extreme weather events such as floods and heatwaves, infectious disease outbreaks resulting from changes observed in patterns of water-borne diseases, poor nutrition due to a reduced food availability and psychosocial effects related to displacement of communities and drought.^2^ Primary care, which provides the first point of contact in the healthcare system, is on the front line in responding to current and future climate change-related health threats.^3^ Owing to its comprehensive, interdisciplinary and longitudinal approach to patients, accounting for the bulk of contacts in the healthcare system, primary care will unavoidably be affected by the health impacts of the changing climate. Further, it has the capacity to directly ease its environmental impact through interventions such as choosing lower carbon options for prescribing where there is clinical equivalence, deprescribing initiatives, use of non-pharmacological alternatives where appropriate, reducing waste and encouraging recycling as well as indirectly reducing the impact of the wider healthcare system.^4^,^6^

Healthcare has a significant impact on greenhouse gas emissions. In the UK, the National Health Service (NHS) contributes 25% of all public sector carbon dioxide emissions, accounting for around 4%–5% of total UK’s carbon emissions.^7 8^ Primary care’s contributions to greenhouse gas emissions derive from the direct delivery of care, staff and patient travel, health and care services commissioned by the NHS, supply chain, infrastructure and prescription of medications, particularly metered-dose inhalers.^9^ The clinical carbon footprint accounts for 74.9% and non-clinical footprint accounts for 20.6% of the total carbon emissions footprint in primary care^10^; general practice is the main provider of primary care in the UK. The Net Zero NHS report recognises primary care as having a key role in attaining net zero but lacks detail on how this should be done, reflecting the need for more research in this area.^11^

The World Organisation of Family Doctors has urged primary care clinicians worldwide to commit to tackling climate change.^12^ Primary care staff are well placed as agents of systemic and individual change.^12^,^14^ Achieving net zero carbon emissions requires leadership, culture and behaviour change at all levels of those working within and using healthcare systems.^15^ Local and national ambitions to attain net zero carbon commitments will require coherent and collaborative action and planning and delivery mechanisms that are locality-based yet nationally aligned.^16^ Although recognition of primary care’s role in addressing climate change is mounting,^7 17^ the distributed organisational structure makes this a particularly difficult challenge. For example, in England, there are around 7000 general practices in 9000 buildings.^18^ Some aspects of change require investment at a national level. For example, NHS Property Services, the landlord for many health centres, is undertaking a 3-year improvement plan aimed at cutting emissions from gas and electricity, installation of smart metres and LED (Light Emitting Diode) lighting and other building upgrades.^7^

This systematic review is part of the GPNET-0 (General Practice Net Zero) study,^19^ which aims to identify whether and how available decarbonisation resources are being used in general practice, what influences this and what is needed to increase their overall impact on decarbonisation. To that end, the review will identify components of processes, behaviours and activities that support climate action in general practice, allowing us to determine the impact of institutional, organisational, professional and individual factors on their implementation and effectiveness. The review will be used to refine an initial programme theory (IPT) (online supplemental additional file 1), as well as inform subsequent phases of GPNET-0 study, including general practice and patient surveys and interviews. Given that decarbonisation actions are likely to be affected by institutional, organisational and individual behavioural factors, as well as contextual factors such as the views and experiences of patients, the review will employ the sociological Normalisation Process Theory (NPT)^20^ and behavioural Theoretical Domains Framework (TDF) theories to structure data collection and analysis.^21^ While the TDF’s domains comprise individual-level factors that influence behaviour, such as knowledge and skills, social factors, and environment and resource factors,^22^ the NPT explores implementation from a sociological perspective that acknowledges that collective activity requires a multitude of interactions among professionals, patients and others.^23^ The NPT is concerned with three core issues—implementation (social organisation of bringing new practices into action), embedding (process through which new practices are incorporated into everyday work) and integration (process by which practices are reproduced).^22^ As such, it has the capacity to identify how relationships between participants may be affected and how interventions may be modified to support these interactions.^24^

Employing the TDF and NPT frameworks together will enhance the comprehensiveness and confidence of our theoretical model by facilitating a systematic identification of cognitive, affective and environmental determinants relevant to the implementation of decarbonising actions within general practice and understanding of the dynamic social processes (facilitators and barriers) that are involved. This combined approach has recently been successfully used to explain evidence-based practice in primary care^25^ and is starting to gain interest in intervention design.^26^

### Research question

How do institutional, organisational, professional and patient factors influence the implementation and sustainability of actions to mitigate the greenhouse gas emissions associated with general practice?

### Objectives

This systematic review is divided into two sequential parts. The first part of the review aims to:

1. Summarise the existing literature on the implementation of decarbonisation actions in general practice.
2. Outline the actions being implemented.
3. Outline the factors influencing decarbonisation.
4. Identify evidence gaps.
5. Identify questions for future research.

The second part of the systematic review will draw on the NPT and the TDF theories to structure the data collected in the core part of the systematic review.

General practice is the main focus of this review as not only is it the main provider of primary care (in the UK), but general practitioners (GPS) and their teams also ‘have specialist knowledge of the areas they serve and the patients they care for and are usually best placed to improve the health and well-being of residents’.^27^

## Methods

The first part of the systematic review will assess general practice carbon reduction activities employing a narrative synthesis approach.^28^,^30^ The second part, which will be a separate research article, will use a framework analysis drawing on the NPT and the TDF coding dictionaries following the method described by Glidewell *et al*.^25^ The review was developed in accordance with the Preferred Reporting Items for Systematic Review and Meta-Analysis guidelines^31^ and is registered on PROSPERO, an international register of systematic reviews.^32^ Any changes to the protocol will be recorded on PROSPERO. The evidence to be included in the review can be described using the PICO (Population, Intervention, Comparison and Outcomes) criteria, as follows.^31^

### Population

The review will include studies that assess the implementation and sustainability of decarbonisation actions in general practice by patients, clinicians (GPs, nurses, pharmacists, physician associates, healthcare assistants), non-clinicians (eg, practice managers, receptionists) and health service policymakers and commissioners.

### Intervention

1. Decarbonisation actions at institutional level (health system/health service).
2. Decarbonisation actions at organisational level (general practice based, with a focus on general practice or equivalent settings).
3. Decarbonisation actions at professional level (clinician and non-clinician).
4. Decarbonisation actions at patient level (individual perspective).

## Outcomes

The outcomes of interest are decarbonisation actions, and factors influencing the adoption, implementation and integration of decarbonisation actions (institutional, organisational, professional, patient factors).

### Study designs

All study designs will be eligible for inclusion, regardless of publication type (eg, journal article, conference publications) and country of study. The Mixed Methods Appraisal Tool (MMAT) will be used to assess the quality of studies. Only English publications will be included, from year 2007 onwards. The selected date coincides with the UN climate change conference, where negotiations on a successor to the Kyoto Protocol began.^33^ Full inclusion and exclusion criteria can be found in Box 1 below.

Box 1 **Inclusion/exclusion criteria**

**Inclusion criteria**

- Any study design.
- Studies set within general practice or data presented that is specific to general practice (or equivalent in non-UK studies).
- Studies that examine institutional, organisational, professional and/or patient factors that influence the implementation and effectiveness of actions to mitigate the greenhouse gas emissions associated with general practice.
- Published in English from 2007 onwards.
- Peer-reviewed and/or grey literature.

**Exclusion criteria**

- Abstract only
- Duplicated texts
- Texts unavailable in English
- Editorials and letters
- Studies within general practice that are specific to community pharmacy, walk-in centres, dental and optometry (eye health) services (or equivalent in non-UK studies).

### Information sources

The following electronic databases will be searched (from 2007 onwards): Medline and Embase (via OvidSP), Web of Science and CINAHL. Similarly, a grey literature search will be conducted on ProQuest.

The automated search will be supplemented by manual backward and forward citation searches of the reference lists of studies included in the review.^34^

### Search strategy

The search strategy was developed through consultation with a librarian with expertise in systematic review searching, employing an iterative process of preliminary searches. The studies to be included will be from 2007 to 29 March 2024, no study design limits will be imposed on the search. Search terms used will be broad and simple in order to capture all potentially relevant studies. The search terms are grouped according to four core concepts, (see draft search strategy in online supplemental additional file 2): (1) setting, (2) implementation, (3) evidence-based practices and (4) subjects. The search terms are based on known studies relating to decarbonisation actions in general practice consulted in the setting up of the GPNET-0 study.

### Data management

The search results will be exported to EndNote (referencing software), where duplicates will be removed electronically, and then exported to Covidence, a systematic review tool designed to support the review process.^35^

### Selection process

All study titles and abstracts will be screened by two independent reviewers with a background in mixed methods research. First, titles and abstracts of articles returned from initial searches will be screened based on the eligibility criteria noted. Studies will be rated ‘yes’ or ‘no’. Articles rated by both reviewers as ‘yes’ or ‘unsure’ will be included in the next phase of study selection.

Second, full texts will be examined in detail and screened for eligibility. If need be, reviewers will seek additional information from study authors to resolve any concerns about eligibility. Where two reviewers disagree and this cannot be resolved by internal discussion, additional members of the research team will be consulted. Reasons for exclusion will be recorded. Third, references of all considered articles will be hand searched to identify any relevant article that may have been missed in the search strategy.

### Data extraction

The researchers will use a data extraction form (see online supplemental additional file 3) to organise the data. Data extraction forms will be piloted and amended as necessary. They will be based on key variables such as study type, sample size, data collection technique and outcomes as well as constructs derived from the TDF and NPT frameworks.

### Quality of individual studies

All included studies will be independently evaluated by two researchers. Quality assessment will be conducted using the MMAT. The MMAT is appropriate for use where included studies use a range of methodologies.^36^

### Synthesis

We will use data from quantitative, qualitative and mixed methods studies.^37^ The synthesis will be conducted in five phases following a deductive and iterative approach,^38^ where a tentative framework of themes and concepts will be used to analyse data. This up-front framework will be developed from elements that have been identified in the IPT (online supplemental additional file 1), the 14 TDF domains and the 4 NPT contracts.

Phase I: Initially, to support the management and summarisation of the data, a spreadsheet will be used to generate a matrix for charting the data. Included studies will be collated and organised, for example, by study design, setting, intervention and outcomes (eg, primary vs secondary). An additional table will be created to map the identified interventions according to activity type in order to uncover areas where decarbonisation interventions are lacking so as to be better positioned to offer recommendations about where future implementation studies are needed. Phase II will involve the tabulation of study results. This will be done to identify patterns across the studies, moving beyond the identification, listing and tabulation of results to the exploration of relationships within and across the included studies.

In phase III, content synthesis will be used to codify the data using NVivo V.14. The process will start with open coding using a predefined list of codes to ensure important aspects of the data are not missed. After coding the first few articles, the research team will meet to compare the labels they have applied and agree on a set of codes to apply to all subsequent articles. Identified codes will be grouped together into clearly defined categories, forming an analytical framework.

In phase IV, these codes will be matched to the TDF domains, the NPT constructs and elements of the IPT to allow for a wider consideration of barriers and enablers to decarbonisation actions (across patient related, professional (clinician/non-clinician), organisational and/or institutional factors) and test whether the analytical framework is successful in identifying recurrent themes and revealing any existing gaps.

Finally, in phase V, there will be an assessment of the robustness of the synthesis using the Joanna Briggs Institute critical appraisal tool.^39^ Rigour will be maintained via peer review of coding by two researchers, together with involvement from the wider research team to address discrepancies or disagreements and to support the synthesis of findings. Developing themes will be discussed and agreed at review team meetings with input from the study’s stakeholder advisory group (SAG) and patient and public involvement (PPI) panel, who will also be involved in the interpretation and synthesis of findings. The SAG consists of medical and sustainability experts, practitioners, policymakers and civil servants. The PPI panel is made up of nine members of the public. They were recruited for the study via the National Institute for Health and Care Research-supported website, *People in Research*.^40^

The review’s findings as well as views from the study’s SAG and PPI panel will be used to refine a programme theory that will inform future phases of the GPNET-0 study.^19^ Further, the findings have the potential to inform decarbonisation policy and practices in general practice and reveal any uncertainties or gaps in our understanding of addressing climate change in the healthcare sector.

## Ethics and dissemination

This review did not involve the collection or analysis of any data that was not included in previously published research in the public domain. It, therefore, was exempt from formal ethical review by the University of Warwick Ethics Committee.

## supplementary material

10.1136/bmjopen-2024-087795 online supplemental file 1

## References

[1] Sawyer M, Partnership H (2022). Humber and North Yorkshire ICB.

[2] Blashki G (2007). GPs and the environment. Aust Fam Physician.

[3] England N (2023). Primary care services nhs website: nhs. https://www.england.nhs.uk/get-involved/get-involved/how/primarycare/#:~:text=Primary%20care%20services%20provide%20the,optometry%20(eye%20health)%20services.

[4] Kemple T (2019). Planetary health and primary care: what’s the emergency?. Br J Gen Pract.

[5] Sawyer MT (2022). Carbon footprint of gp practices across humber and north yorkshire ics.

[6] Gonzalez-Holguera J, Gaille M, Del Rio Carral M (2022). Translating Planetary Health Principles Into Sustainable Primary Care Services. Front Public Health.

[7] England N (2020). Delivering a 'Net zero national health service.

[8] Naylor CaA J (2012). Sustainable health and social care, connecting environmental and financial performance.

[9] Tennison I, Roschnik S, Ashby B (2021). Health care’s response to climate change: a carbon footprint assessment of the NHS in England. Lancet Planet Health.

[10] Humber and North Yorkshire I (2023). Green primary care: NHS. https://humberandnorthyorkshire.org.uk/our-work/climate-change-sustainability/green-primary-care.

[11] BMA (2023). British medical association. https://www.bma.org.uk/what-we-do/population-health/protecting-people-from-threats-to-health/more-support-needed-to-help-the-nhs-reach-net-zero.

[12] Haines A (2019). WONCA: wonca global family doctor. https://www.globalfamilydoctor.com/News/InmyViewDeclarationonPlanetaryHealth.aspx.

[13] Wabnitz K-J, Gabrysch S, Guinto R (2020). A pledge for planetary health to unite health professionals in the Anthropocene. Lancet.

[14] Haines A, Floss M (2021). The inverse care law in the Anthropocene epoch. Lancet.

[15] Atwoli L, Baqui AH, Benfield T (2021). Call for emergency action to limit global temperature increases, restore biodiversity, and protect health. BMJ.

[16] Gudde P, Oakes J, Cochrane P (2021). The role of UK local government in delivering on net zero carbon commitments: You’ve declared a Climate Emergency, so what’s the plan?. Energy Policy.

[17] Powell J (2021). The rise of the green general practice. BMJ.

[18] Wilkinson E (2021). How to achieve a net zero carbon NHS during a pandemic. BMJ.

[19] Study G (2024). GPNET-0 study warwick medical school: gpnet-0 study. https://warwick.ac.uk/fac/sci/med/research/hscience/apc/qualityandsafety/gpnet-0.

[20] Finch TL, Rapley T, Girling M (2013). Improving the normalization of complex interventions: measure development based on normalization process theory (NoMAD): study protocol. Implement Sci.

[21] Atkins L, Francis J, Islam R (2017). A guide to using the Theoretical Domains Framework of behaviour change to investigate implementation problems. Implement Sci.

[22] Stewart D, Klein S (2016). The use of theory in research. Int J Clin Pharm.

[23] Currie K, King C, McAloney-Kocaman K (2019). Barriers and enablers to meticillin-resistant Staphylococcus aureus admission screening in hospitals: a mixed-methods study. J Hosp Infect.

[24] Murray E, Treweek S, Pope C (2010). Normalisation process theory: a framework for developing, evaluating and implementing complex interventions. BMC Med.

[25] Glidewell L, Hunter C, Ward V (2022). Explaining variable effects of an adaptable implementation package to promote evidence-based practice in primary care: a longitudinal process evaluation. Impl Sci.

[26] Band R, Bradbury K, Morton K (2017). Intervention planning for a digital intervention for self-management of hypertension: a theory-, evidence- and person-based approach. Impl Sci.

[27] England N (2019). Primary care - nhs england: nhs england. https://www.england.nhs.uk/wp-content/uploads/2019/03/integrated-care-case-study-primary-care.pdf.

[28] Andrews E, Pearson D, Kelly C (2013). Carbon footprint of patient journeys through primary care: a mixed methods approach. Br J Gen Pract.

[29] Pavli A, Loblay V, Rychetnik L (2023). What can we learn from Australian general practices taking steps to be more environmentally sustainable? A qualitative study. Fam Pract.

[30] Fehrer V, Poß-Doering R, Weis A (2023). Climate change mitigation: Qualitative analysis of environmental impact-reducing strategies in German primary care. Eur J Gen Pract.

[31] Moher D, Shamseer L, Clarke M (2015). Preferred reporting items for systematic review and meta-analysis protocols (PRISMA-P) 2015 statement. Syst Rev.

[32] Centre for Reviews and Dissemination (2023). International prospective register of systematic reviews. https://www.crd.york.ac.uk/prospero.

[33] UN (2007). Bali climate change conference. https://unfccc.int/conference/bali-climate-change-conference-december-2007#news.

[34] Pickard Strange M, Booth A, Akiki M (2023). The Role of Virtual Consulting in Developing Environmentally Sustainable Health Care: Systematic Literature Review. J Med Internet Res.

[35] Kellermeyer L, Harnke B, Knight S (2018). Covidence and Rayyan. jmla.

[36] Hong QN, Fàbregues S, Bartlett G (2018). The Mixed Methods Appraisal Tool (MMAT) version 2018 for information professionals and researchers. EFI.

[37] Horswood D, Baker J, Fazel M (2019). School factors related to the emotional wellbeing and resettlement outcomes of students from refugee backgrounds: protocol for a systematic review. Syst Rev.

[38] Popay J, Roberts HM, Sowden AJ (2009). Guidance on the conduct of narrative synthesis in systematic reviews.

[39] Aromataris EF, Godfrey C, Holly C, Aromataris EMZ (2020). JBI manual for evidence synthesis: JBI.

[40] PiR (2024). Opportunities for public involvement in nhs, public health and social care research. https://www.peopleinresearch.org.

