# Supplementary material for: Implementation of decarbonisation actions in general practice: a systematic review and narrative synthesis protocol
Source: BMJ Open. 2024 Sep 16;14(9):e087795. doi: 10.1136/bmjopen-2024-087795 (PMC11409232; doi:10.1136/bmjopen-2024-087795)
Supplement: online supplemental file 1 [file bmjopen-14-9-s001.pdf]

Appendices

Additional file 1: Initial Programme Theory (IPT) – hypothesised from Primary Care Decarbonisation Resources, Literature and Stakeholder and Researcher Insights

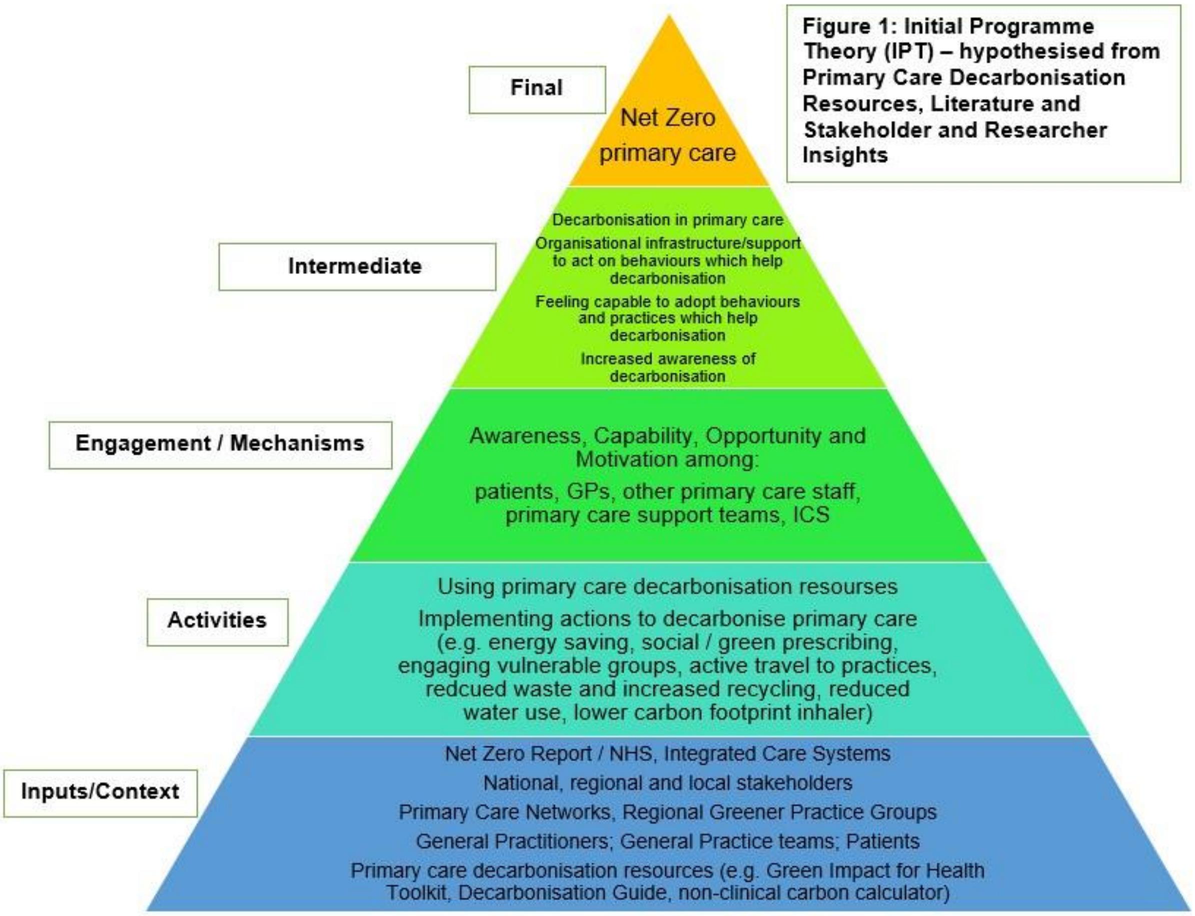

Additional file 2

Piloted search query with key search terms and their combination to be used

Concept	Description	Search Terms
**1)**	Setting	"General practice" OR "primary care" OR "family practice" OR "family clinic" OR "family medicine" OR "community health" OR "medical centre" OR "Primary healthcare" OR "Primary health care"
**2)**	Implementation	"Greenhouse gas*" OR "GHG"OR "Net zero" OR "Net-zero" OR "climate change" OR "carbon emissions" OR "*carbon footprint" OR "environmental sustainability"
**3)**	Intervention	"implement*" OR "strateg*" OR "action*" OR "intervention*" OR "policies" OR "policy" OR "solution*" OR "plan"
**4)**	Subjects	"staff" OR "patient*" OR "team*" OR "employee*"

Search term examples with key mesh terms:

	**MEDLINE**
1	("General practice" or "primary care" or "family practice" or "family clinic" or "family medicine" or "community health" or "medical centre" or "Primary healthcare" or "Primary health care").mp.
2	limit 1 to (english language and yr="2007 - 2023")
3	("Greenhouse gas*" or "GHG" or "Net zero" or "Net-zero" or "climate change" or "carbon emissions" or "*carbon footprint" or "environmental sustainability").mp.
4	limit 3 to (english language and yr="2007 - 2023")
5	2 and 4
6	("implement*" or "strateg*" or "action*" or "intervention*" or "policies" or "policy" or "solution*" or "plan").mp.
7	limit 6 to (english language and yr="2007 - 2023")
8	("staff" or "patient*" or "team*" or "employee*").mp.
9	limit 8 to (english language and yr="2007 - 2023")
10	7 and 9
11	5 and 10

Web of Science

(“General practice” OR “primary care” OR “family practice” OR “family clinic” OR “family medicine” OR “community health” OR “medical centre” OR “Primary healthcare” OR “Primary health care”) (Topic) and (“Greenhouse gas*” OR “GHG” OR “Net zero” OR “Net-zero” OR “climate change” OR “carbon emissions” OR “*carbon footprint” OR “environmental sustainability”) (Topic) and (“implement*” OR “strateg*” OR “action*” OR “intervention*” OR “policies” OR “policy” OR “solution*” OR “plan") (Topic) and (“staff” OR “patient*” OR “team*” OR “employee*”) (Topic)

ProQuest

noft("General practice" OR "primary care" OR "family practice" OR "family clinic" OR "family medicine" OR "community health" OR "medical centre" OR "Primary healthcare" OR "Primary health care") AND noft("Greenhouse gas*" OR "GHG" OR "Net zero" OR "Net-zero" OR "climate change" OR "carbon emissions" OR "carbon footprint" OR "environmental sustainability") AND noft("implement*" OR "strateg*" OR "action*" OR "intervention*" OR "policies" OR "policy" OR "solution*" OR "plan") AND noft("staff" OR "patient*" OR "team*" OR "employee*")

Source type: Blogs, Podcasts, & Websites, Books, Conference Papers & Proceedings, Dissertations & Theses, Magazines, Reports
Language: English

CINAHL

(“General practice” OR “primary care” OR “family practice” OR “family clinic” OR “family medicine” OR “community health” OR “medical centre” OR “Primary healthcare” OR “Primary health care”) AND (“Greenhouse gas*” OR “GHG” OR “Net zero” OR “Net-zero” OR “climate change” OR “carbon emissions” OR “*carbon footprint” OR “environmental sustainability”) AND (“implement*” OR “strateg*” OR “action*” OR “intervention*” OR “policies” OR “policy” OR “solution*” OR “plan”) AND (“staff” OR “patient*” OR “team*” OR “employee*”)

Limiters - Publication Date: 20070101-20231231; English Language; Language: English
Expanders - Apply equivalent subjects
Search modes - Find all my search terms

Additional file 3

Data extraction form			
**TDF Domains**	Structure	**NPT constructs**	Structure
Knowledge	Patient	Coherence (intervention)	Patient
	Professional		Professional
	Organisational		Organisational
	Institutional		Institutional
Skills	Patient	Cognitive participation (commit to engage)	Patient
	Professional		Professional
	Organisational		Organisational
	Institutional		Institutional
Social/professional role & identity	Patient	Collective action	Patient
	Professional		Professional
	Organisational		Organisational
	Institutional		Institutional
Beliefs (about capabilities)	Patient	Reflexive monitoring (appraise actions)	Patient
	Professional		Professional
	Organisational		Organisational
	Institutional		Institutional
Optimism	Patient	**IPT**	
	Professional	Inputs (e.g non-clinical carbon calculator, green impact for health toolkit)	Patient
	Organisational		Professional
	Institutional		Organisational
Beliefs about consequences	Patient		Institutional
	Professional	Activities (e.g active travel, water use)	Patient
	Organisational		Professional
	Institutional		Organisational
Reinforcement	Patient		Institutional
	Professional	Mechanisms (e.g. awareness, motivation, capability)	Patient
	Organisational		Professional
	Institutional		Organisational
Intentions	Patient		Institutional

	Professional	Intermediate (e.g. increased awareness, increased motivation)	Patient
	Organisational		Professional
	Institutional		Organisational
Goals	Patient		Institutional
	Professional	Final (net zero)	Patient
	Organisational		Professional
	Institutional		Organisational
Memory	Patient		Institutional
	Professional		
	Organisational		
	Institutional		
Attention and decision processes	Patient		
	Professional		
	Organisational		
	Institutional		
Environmental context and resources	Patient		
	Professional		
	Organisational		
	Institutional		
Social influences	Patient		
	Professional		
	Organisational		
	Institutional		
Emotion	Patient		
	Professional		
	Organisational		
	Institutional		
Behavioural regulation	Patient		
	Professional		
	Organisational		
	Institutional		

**Additional file 3**: PRISMA-P (Preferred Reporting Items for Systematic review and Meta-Analysis Protocols) protocol			
PRISMA-P 2015 checklist			
Section and topic	Item No	Checklist item	Page #
**ADMINISTRATIVE INFORMATION**			
Title:			1
Identification		Identify the report as a protocol of a systematic review	1
Update		If the protocol is for an update of a previous systematic review, identify as such	N/A
Registration		If registered, provide the name of the registry (PROSPERO # CRD42023470889)	2
Authors:			
Contact		Provide name, institutional affiliation, e-mail address of all protocol authors; provide physical mailing address of corresponding author	8-9
Contributions		Describe contributions of protocol authors and identify the guarantor of the review	10
Amendments		If the protocol represents an amendment of a previously completed or published protocol, identify as such and list changes; otherwise, state plan for documenting important protocol amendments	N/A
Support:			
Sources		Indicate sources of financial or other support for the review	7-8
Sponsor		Provide name for the review funder and/or sponsor	7
Role of sponsor or funder		Describe roles of funder(s), sponsor(s), and/or institution(s), if any, in developing the protocol	N/A
**INTRODUCTION**			
Rationale		Describe the rationale for the review in the context of what is already known	2-3
Objective		Provide an explicit statement of the question(s) the review will address with reference to participants, interventions, comparators, and outcomes (PICO)	4
**METHODS**			
Eligibility criteria		Specify the study characteristics (such as PICO, study design, setting, time frame) and report characteristics (such as years considered, language, publication status) to be used as criteria for eligibility for the review	4-5
Information sources		Describe all intended information sources (such as electronic databases, contact with study authors, trial	5

		registers or other grey literature sources) with planned dates of coverage.	
Search strategy		Present draft of search strategy to be used for at least one electronic database, including planned limits, such that it could be repeated	Appendices
Study records:			
Data management		Describe the mechanism(s) that will be used to manage records and data throughout the review	6
Selection process		State the process that will be used for selecting studies (such as two independent reviewers) through each phase of the review (that is, screening, eligibility and inclusion in meta-analysis)	6
Data collection		Describe planned method of extracting data from reports (such as piloting forms, done independently, in duplicate), any processes for obtaining and confirming data from investigators	6-7
Data items		List and define all variables for which data will be sought (such as PICO items, funding sources), any pre-planned data assumptions and simplifications	4-5
Outcomes and prioritization		List and define all outcomes for which data will be sought, including prioritization of main and additional outcomes, with rationale	5
Risk of bias in individual studies		Describe anticipated methods for assessing risk of bias of individual studies, including whether this will be done at the outcome or study level, or both; state how this information will be used in data synthesis	6
Data synthesis		Describe criteria under which study data will be quantitatively synthesised	N/A
		If data are appropriate for quantitative synthesis, describe planned summary measures, methods of handling data and methods of combining data from studies, including any planned exploration of consistency (such as I, Kendall's τ)	N/A
		Describe any proposed additional analyses (such as sensitivity or subgroup analyses, meta-regression)	N/A
		If quantitative synthesis is not appropriate, describe the type of summary planned	6-7
Meta-bias(es)		Specify any planned assessment of meta-bias(es) (such as publication bias across studies, selective reporting within studies	N/A
Confidence in cumulative evidence		Describe how the strength of the body of evidence will be assessed (such as GRADE)	N/A
*N/A= Not applicable			
Adapted and modified from: Shamseer L, Moher D, Clarke M, Ghersi D, Liberati A, Petticrew M, Shekelle P, Stewart L, PRISMA-P Group. Preferred reporting items for			

systematic review and meta-analysis protocols (PRISMA-P) 2015: elaboration and explanation. BMJ. 2015 Jan 2;349(jan02 1):g7647	
