# Supplementary material for: Implementation of decarbonisation actions in general practice: a systematic review and narrative synthesis protocol
Source: BMJ Open. 2024 Sep 16;14(9):e087795. doi: 10.1136/bmjopen-2024-087795 (PMC11409232; doi:10.1136/bmjopen-2024-087795)
Supplement: Supplementary file 2 [file bmjopen-2024-087795.reviewer_comments.pdf]

PEER REVIEW HISTORY

BMJ Open publishes all reviews undertaken for accepted manuscripts. Reviewers are asked to complete a checklist review form and are provided with free text boxes to elaborate on their assessment. These free text comments are reproduced below.

ARTICLE DETAILS

**TITLE (PROVISIONAL)**	Implementation of decarbonisation actions in general practice – a systematic review and narrative synthesis protocol
**AUTHORS**	Karaba, Florence\; Nunes, Ana Raquel; Geddes, Olivia; Atherton, Helen; Dahlmann, Frederik; Eccles, Abi; Gregg, Michael; Spencer, Rachel; Twohig, Helen; Dale, Jeremy

VERSION 1 - REVIEW

**REVIEWER NAME**	Davies, Jessica
**REVIEWER AFFILIATION**	Austin Health, Anaesthesia
**REVIEWER CONFLICT OF INTEREST**	None
**DATE REVIEW RETURNED**	10-May-2024

**GENERAL COMMENTS**	This is a nicely written protocol for which I have very little feedback to contribute. The topic is of relevance and the methodology appears to be well considered, thorough and the various tools and frameworks available to enhance the rigour of the review have been considered and utilised appropriately. I look forward to reading the review!

**REVIEWER NAME**	John, Joseph
**REVIEWER AFFILIATION**	University of Exeter Medical School
**REVIEWER CONFLICT OF INTEREST**	None
**DATE REVIEW RETURNED**	29-May-2024

**GENERAL COMMENTS**	General comments:<br>The aim is a very important one. It is great to see that this review is being undertaken. It is clearly being delivered by a team who are very experienced in primary care research and evidence synthesis.<br><br>In my opinion the protocol is suitable for publication, and I just have a few suggestions that the authors might want to consider prior to publication.<br><br>Research question:<br>I wonder whether an additional important question that this review could answer could be:<br>“Where is there an absence of evidence describing the implementation of mitigation actions for important known GHG hotspots in primary care?<br>This could be an important way to frame your findings to understand whether the implementation research carried out to date is in areas of the greatest importance to our net zero targets. Your search

	criteria would not necessarily require modification to be able to generate understanding about this additional question.<br><br>Information sources:<br>I expect the consultations with decarbonisation experts in primary care will be a particularly important part of the information acquisition. Are the inclusion/exclusion criteria sufficiently broad to allow potential information sources described by experts to be included? I'm thinking particularly about the sort of content that might be written up or discussed online through grassroots networks. I appreciate that you might feel that such information sources would be insufficiently rigorous to be included in the review, and therefore this might not be appropriate.<br><br>Objectives/outcomes:<br>Factors relevant to successful implementation of a particular type of decarbonisation strategy might differ by institution/organisation/individual. Do you intend to analyse such differences?<br><br>Effectiveness of studied interventions:<br>In your analysis do you intend to understand what the potential beneficial effects of the studied interventions might be in terms of meeting our net zero targets? And will you comment on the distribution of interventions in relation to the spectrum of activities required to achieve meaningful progress towards net zero? For example, if all studies looked at a narrow range of interventions with modest potential to reduce overall primary care GHG emissions, you might be able to make recommendations about where future implementation studies are most urgently warranted. This point relates to my comment above about research questions.

VERSION 1 – AUTHOR RESPONSE

Reviewer 1

Comment: This is a nicely written protocol for which I have very little feedback to contribute. The topic is of relevance and the methodology appears to be well considered, thorough and the various tools and frameworks available to enhance the rigour of the review have been considered and utilised appropriately. I look forward to reading the review!

Response: The authors thank the reviewer for the encouraging comments and positive feedback. We look forward to submitting the systematic review paper.

Reviewer 2

Comment: The aim is a very important one. It is great to see that this review is being undertaken. It is clearly being delivered by a team who are very experienced in primary care research and evidence synthesis.

In my opinion the protocol is suitable for publication, and I just have a few suggestions that the authors might want to consider prior to publication.

Response: The authors thank the reviewer for the encouraging comments and positive feedback. We look forward to submitting the systematic review paper.

Research question

Comment: I wonder whether an additional important question that this review could answer could be: “Where is there an absence of evidence describing the implementation of mitigation actions for important known GHG hotspots in primary care? This could be an important way to frame your findings to understand whether the implementation research carried out to date is in areas of the greatest importance to our net zero targets. Your search criteria would not necessarily require modification to be able to generate understanding about this additional question.

Response: Thank you very much for your comment. The objectives of the systematic review have been rewritten to address the comment. The following text has been added:

"**Objectives:** To summarise existing literature on the implementation of decarbonisation actions in general practice, to outline the actions being implemented, factors influencing decarbonisation, identify evidence gaps and questions for future research."

Information sources

Comment: I expect the consultations with decarbonisation experts in primary care will be a particularly important part of the information acquisition. Are the inclusion/exclusion criteria sufficiently broad to allow potential information sources described by experts to be included? I’m thinking particularly about the sort of content that might be written up or discussed online through grassroots networks. I appreciate that you might feel that such information sources would be insufficiently rigorous to be included in the review, and therefore this might not be appropriate.

Response: Thank you for your comment and suggestion. The systematic review protocol and paper are part of a larger research project and consultations with climate change stakeholders in general practice will be undertaken as part of the research in other work packages. These will include views on how local, regional and national plans and strategies are aimed at decarbonising general practice and achieving NHS net zero targets. We will work with our public and patient panel, advisory and stakeholder groups’ to identify relevant stakeholders with a broad range of roles.

Regarding the inclusion of content from non-scholarly sources, the inclusion criteria allow for all study designs regardless of publication type, both peer-reviewed and grey literature. This criterion ensures that the referred content would be eligible for inclusion if it meets the other criteria. The ProQuest database was used to search grey literature, including blogs, newspapers, websites, reports, working papers, etc.

Objectives/outcomes:

Comment: Factors relevant to successful implementation of a particular type of decarbonisation strategy might differ by institution/organisation/individual. Do you intend to analyse such differences?

Response: Thank you for this comment. We will analyse these differences. The objectives of the systematic review are:

“To summarise existing literature on the implementation of decarbonisation actions in general practice, to outline the actions being implemented, factors influencing decarbonisation, identify evidence gaps and questions for future research.”

Effectiveness of studied interventions:

Comment: In your analysis do you intend to understand what the potential beneficial effects of the studied interventions might be in terms of meeting our net zero targets? And will you comment on the distribution of interventions in relation to the spectrum of activities required to achieve meaningful

progress towards net zero? For example, if all studies looked at a narrow range of interventions with modest potential to reduce overall primary care GHG emissions, you might be able to make recommendations about where future implementation studies are most urgently warranted. This point relates to my comment above about research questions.

Response: Thank you for your comments. Within the broader research project, we will seek to understand what the potential beneficial effects of the studied interventions might be in terms of contributing to decarbonising general practice. In our analysis, we will include all the interventions identified in the included studies according to activity type to uncover areas where decarbonisation interventions are lacking as to be better positioned to offer recommendations about where future implementation studies are needed. We have added this point to the section on synthesis.

VERSION 2 - REVIEW

**REVIEWER NAME**	John, Joseph
**REVIEWER AFFILIATION**	University of Exeter Medical School
**REVIEWER CONFLICT OF INTEREST**	none
**DATE REVIEW RETURNED**	29-Jul-2024

**GENERAL COMMENTS**	Thank you for your responses. I look forward to reading the results of the review.
